# Supramolecular structure of dietary fat in early life modulates expression of markers for mitochondrial content and capacity in adipose tissue of adult mice

**DOI:** 10.1186/s12986-017-0191-5

**Published:** 2017-06-12

**Authors:** Andrea Kodde, Eline M. van der Beek, Esther Phielix, Eefje Engels, Lidewij Schipper, Annemarie Oosting

**Affiliations:** 1 Earl Life Nutrition Division, Nutricia Research, P.O. Box 80141, 3508 TC Utrecht, The Netherlands; 2 Department of Pediatrics, University Medical Centre Groningen, Groningen, The Netherlands; 3 Department of Human Biology, Maastricht University, Maastricht, The Netherlands

**Keywords:** Postnatal programming, White adipose tissue, Mitochondrial content, Oxidative capacity, Lipid droplet structure

## Abstract

**Background:**

Previous studies have shown that early life nutrition can modulate the development of white adipose tissue and thereby affect the risk on obesity and metabolic disease later in life. For instance, postnatal feeding with a concept infant milk formula with large, phospholipid coated lipid droplets (Concept, Nuturis®), resulted in reduced adiposity in adult mice. The present study investigated whether differences in cell energy metabolism, using markers of mitochondrial content and capacity, may contribute to the observed effects.

**Methods:**

C57Bl/6j male mice were exposed to a rodent diet containing the Concept (Concept) or standard (CTRL) infant milk formula from postnatal day 16 until postnatal day 42, followed by a western style diet challenge until postnatal day 98. Markers for mitochondrial content and capacity were analyzed in retroperitoneal white adipose tissue and gene expression of metabolic markers was measured in both retroperitoneal white adipose tissue and *muscle tibialis (M. tibialis)* at postnatal day 98.

**Results:**

In retroperitoneal white adipose tissue, the Concept group showed higher citrate synthase activity and mitochondrial DNA expression compared to the CTRL group (*p* < 0.05). In addition, protein expression of mitochondrial cytochrome c oxidase subunit I of the oxidative phosphorylation pathway/cascade was increased in the Concept group compared to CTRL (*p* < 0.05). In the *M. tibialis,* gene expression of uncoupling protein 3 was higher in the Concept compared to the CTRL group. Other gene and protein expression markers for mitochondrial oxidative capacity were not different between groups.

**Conclusion:**

Postnatal feeding with large, phospholipid coated lipid droplets generating a different supramolecular structure of dietary lipids enhances adult gene and protein expression of specific mitochondrial oxidative capacity markers, indicative of increased substrate oxidation in white adipose tissue and skeletal muscle. Although functional mitochondrial capacity was not measured, these results may suggest that adaptations in mitochondrial function via early feeding with a more physiological structure of dietary lipids, could underlie the observed beneficial effects on later life adiposity.

**Electronic supplementary material:**

The online version of this article (doi:10.1186/s12986-017-0191-5) contains supplementary material, which is available to authorized users.

## Background

Obesity is a global health threat and increasingly emerges at a young age [1]. Early onset obesity is associated with metabolic disease, including diabetes and cardiovascular disease [1]. Increased energy intake and decreased physical activity are well-established lifestyle risk factors in obesity [2]. When energy intake exceeds energy expenditure, the surplus energy is stored in white adipose tissue (WAT) resulting in increased adipocyte size (hypertrophy) and eventually increased adipocyte numbers (hyperplasia) [3–5]. When WAT fails to store the excessive energy, fat accumulates ectopically in the liver and skeletal muscle, an important step in the development of type 2 diabetes (T2D) [6].

A mismatch between storage of fat in the form of triglycerides and oxidation of fatty acids (FA) in the mitochondria is an important feature in both obesity and T2D [7]. Moreover, compromised mitochondrial function in skeletal muscle [8–10], as well as in liver and WAT [11, 12] evaluated using functional measurements, is a well know feature seen in obesity and T2D. In line with these observations, gene expression of a subset of genes coding for mitochondrial proteins (i.e. pyruvate dehydrogenase, cytochrome C, carnitinepalmitoyl-transferase I (*Cpt1*) and the uncoupling proteins (*Ucp*s)) were lower in WAT of transgenic diabetic mice models and in high fat diet (HFD)-induced insulin-resistant mice [11, 13]. Taken together, these data strongly indicate a pivotal role of mitochondrial energy metabolism in WAT in obesity and T2D.

The development of WAT starts in the third trimester of gestation and continues throughout childhood and adolescence [3]. Nutrition (excess or scarcity) during pregnancy and infancy has a substantial effect on adipose tissue development. For instance, maternal exposure to a HFD during pregnancy and lactation resulted in increased adiposity in rodent offspring [14, 15]. This indicates that the nutrition in early life programs adiposity in adulthood.

Observational studies have demonstrated a moderate but consistent association between breastfeeding (duration) and reduced prevalence of later life overweight and obesity [16, 17]. Studies from our lab suggest that the supramolecular structure of lipids in milk may contribute to these beneficial effects [18–20]. We have shown that early postnatal exposure to a diet containing a concept IMF with large lipid droplets coated by phospholipids (Concept, Nuturis®) reduced body fat accumulation in adolescence and early adulthood in a mouse model for nutritional programming [18, 19]. The reduced adult fat mass was associated with reduced adipocyte size and altered adipocyte function as well as improved metabolic health [19]. This provides clear evidence that early life dietary lipid supramolecular structure may effectively program metabolic health later in life.

Nutrition during fetal and early postnatal life has been shown to affect mitochondrial content and oxidative capacity throughout life [21–24]. For instance, hepatic enzyme activity of the mitochondrial oxidative phosphorylation was decreased in offspring of dams fed a HFD during pregnancy and lactation [21]. Thus, differences in mitochondrial development and function may underlie programming of adiposity and metabolic health later in life.

In the current study, we investigate if postnatal exposure to large, phospholipid coated lipid droplets affects markers for mitochondrial content and oxidative capacity in WAT. We hypothesize that the supramolecular lipid structure affects the expression of genes involved in the energy utilization in both WAT and skeletal muscle and the expression of markers for mitochondrial content and oxidative capacity in WAT.

## Methods

### Animals and study design

**Fig. 1:**
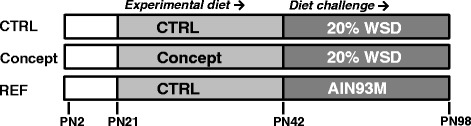
Study design; WSD: western style diet; PN: postnatal day

All experimental procedures were approved by an external, independent Animal Experimental Committee (DEC consult, Soest, The Netherlands) and complied with the principles of good laboratory animal care following the EU-directive for the protection of animals used for scientific purposes. C57Bl/6jOlaHsd mice (Harlan, Horst, The Netherlands) were kept at the animal facility of Wageningen University and Research Centre under a 12 h light – 12 h dark cycle (lights on at 06:00 h). Room temperature and humidity were kept at a constant level (21 ± 2 °C and 50 ± 5% respectively) and standard cage enrichment was added to allow mice to build a nest. Food and water were available ad libitum during the entire experimental period, except for an 8 h fastening period before dissection. Breeders were time-mated and female mice were provided with the American Institute of Nutrition-93G synthetic diet (AIN93G) [25] during pregnancy and lactation. At postnatal day 2 (PN2), litters were randomly cross-fostered to reduce between-litter variability and avoid litter effects, and culled to four male and two female pups per dam. On PN16, litters were randomly assigned to either the *i.* control (CTRL), *ii.* Concept or *iii.* Non-challenged reference group (REF), resulting in *n* = 12 male pups per experimental group (3 litters per group). The Concept group was fed the Concept-IMF based diet and CTRL and REF group were fed a control-IMF based diet from PN16 until PN42 (Fig. 1). Male littermates were housed in pairs after weaning at PN21 and continued their respective IMF-based diets. From PN42 until PN98, Concept and CTRL group were challenged with a western style diet containing 20 *w*/w % fat (WSD, Fig. 1). The REF group was fed AIN93M [25] from PN42 until PN98 and was included as a non-challenged reference to compare to the effects of the WSD challenge (CTRL group). Body weight was measured twice a week as of the period of weaning and body composition was monitored by DEXA using a PIXImus imager (GE Lunar, Madison, WI, USA) under general anesthesia (isoflurane/N_2_O/O_2_). At PN98, mice were fasted for 8 h, subjected to terminal anesthesia (isoflurane/N_2_O/O_2_) and dissected. *M.tibialis* and retroperitoneal (RP) WAT depots (as proxy for visceral WAT) were weighed, snap frozen and stored at −80 °C. Epididymal (EPI) WAT depots were weighed and immediately used for fat cell analysis, as RP WAT depots were too small to use for all analyses.

### IMF production

The CTRL IMF was produced according to a Standard stage 1 IMF recipe and processing procedure (Nutricia Research, Utrecht, the Netherlands). For the Concept IMF powder 0.5 g/l phospholipids of bovine milk origin (SM2, Corman Food industry, Goé, Belgium) was added and processing procedure was adjusted as described previously [19, 26]. The mode diameter of the lipid droplets in the Concept IMF was 2.924 μm versus 0.267 μm in the CTRL IMF.

### Experimental diets

Control and Concept IMF-based diets were semi-synthetic and contained 28.2 *w*/w% control or Concept IMF powder, respectively. The diets were complemented with protein and carbohydrates to establish macronutrient composition appropriate for normal growth and development in rodents and match a macronutrient composition according to AIN93G standards (Table 1). The macronutrient composition of CTRL and Concept diet was similar except for phospholipid (0.2 versus 1.44 g/kg, respectively) content. The moderate WSD consisted of 20% (*w*/w) fat of which 17% (*w*/w) lard, 3% (*w*/w) soy oil and 0.1% (*w*/w) cholesterol (Table 1).

**Table 1:** Composition of the early programming diets and the WSD diet

Diet		Programming diet		WSD
		CTRL	Concept	
Carbohydrates	(g/kg)	646	646	520
Sugars^a^	(g/kg)	236	236	70
Polysaccharides^b^	(g/kg)	410	410	450
Protein	(g/kg)	179	179	180
Fat	(g/kg)	70	70	200
Saturated Fatty Acids (SFA)	(g/kg)	30	30	81,2
Mono Unsaturated Fatty Acids (MUFA)	(g/kg)	28	28	84,9
Poly Unsaturated Fatty Acids (PUFA)	(g/kg)	12	12	32,7
LA/ALA ratio		5.5	5.5	14,9
Phospholipids^c^	(g/kg)	0.2	1.44	-
Cholesterol	(g/kg)	-	-	1.0
Fiber	(g/kg)	47.5	47.5	50
Vitamin & mineral mix	(g/kg)	45	45	45

^a^Total sugars, including lactose, glucose and sucrose

^b^Including starch and maltodexintrin

^c^Phospholipids derived from bovine milk, as a and b

### Fat cell analysis

Fresh EPI WAT depots were used to determine cell size distribution according to the optical method of DiGirolamo and Fine [27] and Hirsch and Gallian [28] as described in detail previously [19].

### Gene expression

RNA from RP WAT and *m.tibialis* were isolated using Trizol®/chloroform (Invitrogen, Breda, The Netherlands) and purified with a RNeasy Mini Kit (Qiagen Benelux b.v., Zwijndrecht, The Netherlands) including a DNase treatment with a RNase-free DNase Set (Qiagen Benelux b.v., Zwijndrecht, The Netherlands) as previously described [29]. Quantity and quality of the RNA samples was analysed with the Nanodrop 2000 (Thermo Scienctific, Breda, The Netherlands) and the Bioanalyzer (Agilent, Santa Clara, USA). Samples with a RIN below 8.0 and a 260/280 ratio below 1.9 were excluded from analyses. cDNA was synthesized with the iScript™ cDNA synthese kit (Bio-Rad, Veenendaal, The Netherlands) according to manufacturer instructions. 9,4 and 25 ng cDNA was used as input for each Q-PCR reaction of the RP WAT and *m.tibialis* samples, respectively. 5× Hot FirePol Evagreen® qPCR mix Plus (Bio-Connect, Huissen, The Netherlands) was used according to manufacturer instructions and qPCR was performed with a 7900HT Fast Real Time PCR System (Applied Biosystems, Bleiswijk, The Netherlands). Acetyl-CoA carboxylase 1 (*Acc1*), adipose triglyceride lipase (*Atgl*), fatty acid transfer protein/cluster of differentiation 36 (*Cd36*), Cell death-inducing DNA fragmentation factor, alpha subunit-like effector A (*Cidea*)*, Cpt1α*, fatty acid binding protein 4 (*Fabp4*), fatty acid synthase (*Fasn*), fatty acid transfer protein 1 (*Fatp1*), glucose transporter 4 (*Glut4*), glycerol-3-phosphate acyltransferase (*Gpat*), hexokinase II (*HkII*), hormone sensitive lipase (*Hsl*), lipoprotein lipase (*Lpl*), pyruvate dehydrogenase kinase 4 (*Pdk4*), peroxisome proliferator activated receptor α (*Pparα*), stearoyl-Coenzyme A desaturase 1 (*Scd1*), sterol regulatory element binding protein 1c (*Srebp1c*), *Ucp1* and *Ucp3* mRNA expression were measured in RP WAT. *Cpt1α*, *Glut4*, *Pdk4* and *Ucp3* gene expression were measured in *m.tibialis*. See Table 2 for the complete list of primers. RP WAT qPCR data was normalized using the method of relative normalization as described by Hellemans et al. [30] and performed with qbase^+^ (Biogazelle, Genth, Belgium). In short, gene expression of genes of interest was analyzed relative to mean expression of multiple reference genes and scaled to the expression of the CTRL group. Four reference genes were used for the RP WAT: ribosomal protein L19 (*Rpl19*) and S29 (*Rps29*), 18S ribosomal RNA (*18SrRNA*) and Calnexin (*Canx*). Rpl19, Rps29 and 18SrRNA were used as reference genes for the *M. tibialis*.

**Table 2:** Primer sequences

Gene name	NCBI Reference number	Forward primer	Reverse primer
Genes of interest:			
*Acc1*	NM_133360.2	tggtgcagaggtaccgaagtg	cgtagtggccgttctgaaact
*Atgl*	NM_025802.3	atccgcttgttggagtggct	ctcttggccctcatcaccag
*Cd36*	NM_001159558.1	attgcgacatgattaatggcac	gatagacctgcaaatgtcagaggaa
*Cidea*	NM_007702.2	aggccgtgttaaggaatctg	cccagtactcggagcatgta
*Cpt1α*	NM_013495.2	gcccatgttgtacagcttcc	tcatcagtggcctcacagac
*Fabp4*	NM_024406.2	tcataaccctagatggcggg	ttccaccaccagcttgtcac
*Fas*	NM_007988.3	gctgggctctatggattaccc	ggtccattgtgtgtgcctgc
*Fatp1*	NM_011977.3	attgtggtgcacagcaggtacta	tggtagagtggcaggcagtca
*Glut4*	NM_009204.2	cggatgctatgggtccttacg	aacgtccggcctctggttt
*Gpat*	NM_008149.3	tgtccacaacttcagcggtc	cagcgtacacggcaacgtt
*HkII*	NM_013820.3	aagacgataaggacggaattcaga	gagcgcgtggacacaatct
*Hsl*	NM_010719.5	cctgcttggttcaactggaga	tcactccataggctgctgcc
*Lpl*	NM_008509.2	cacttgtcatctcattcctggatt	ccgttaccgtccatccatg
*Pdk4*	NM_013743.2	aagagctggtatatccagagcctg	ttgaccagcgtgtctacaaactc
*Pparα*	NM_011144.6	agtgccctgaacatcgagtgt	ttcgccgaaagaagccctta
*Scd1*	NM_009127.4	cacctgcctcttcgggatttt	gcagccgtgccttgtaagttc
*Srebp1c*	NM_011480.3	ccggctattccgtgaacatc	caagggcatctgagaactccc
*Ucp1*		assay ID qMmuCID0005832	
*Ucp3*	NM_009464.3	aacgctcccctaggcaggta	gcagaaaggagggcacaaatc
Reference genes:			
*Canx*	NM_007597.3	agagctcagcctggatcaattc	ttgtagtcctctccacacttatctgg
*Rpl19*	NM_009078.2	ttgcctctagtgtcctccgc	cttcctgatctgctgacggg
*Rps29*	NM_009093.2	agtcacccacggaagttcgg	gtccaacttaatgaagcctatgtcctt
*18SrRNA*	NR_003278.1	cgattccgtgggtggtggtg	catgccagagtctcgttcgttatc
DNA primers			
*Nd1*	NC_005089.1	accaatacgccctttaacaac	aatgggtgtggtattggtagg
*Lpl*	NM_008509	tcctgatgacgctgattttg	atgtcaacatgccctactgg

### Enzyme activity

Citrate synthase (CS) and Hydroxyacyl-CoA dehydrogenase (HAD) activity were measured as described elsewhere [31]. In short, RP WAT samples were homogenized and dissolved in sucrose-Tris-EDTA buffer (250 mM Sucrose, 10 mM Tris, 2 mM EDTA, pH 7.4). CS activity was analyzed by adding sample to reaction reagent (100 mM Tris, 100 μM DTNB, 50 μM acetyl-CoA, pH 8.0) and start reagent (50 mM Oxaloacetic acid), in the proportion 1:50:1, followed by a kinetic reading (421 nm, 37 °C). HAD activity was analyzed by adding sample to reaction reagent (100 mM tetra-sodiumpyrophosphate, 250 μM NADH, pH 7.3) and start reagent (5 mM Acetoacetyl-CoA), in the proportion 1:10:1, followed by a kinetic reading (340 nm, 37 °C).

### OXPHOS protein expression

Protein expression of 5 subunits of the oxidative phosphorylation (OXPHOS) was measured as previously described [32]. Briefly, RP WAT samples were homogenized and dissolved in Radio-Immunoprecipitation Assay buffer (RIPA, Fisher Scientific, Landsmeer, The Netherlands) with protease inhibitor cocktail (Roche diagnostics, Almere, the Netherlands). Per sample 15 μg was used for gel electrophoreses with a 4–15% gradient and transferred to a PVDF membrane with a Trans-Blot® Turbo™ Blotting System using the Trans-Blot® Turbo™ Midi PVDF Transfer pack (Bio-Rad, Veenendaal, The Netherlands). OXPHOS protein expression was determined using the Mito-Profile® Total OXPHOS rodent western blot antibody cocktail (Abcam, Cambridge, UK) with ECL anti mouse IgG (Fisher Scientific, Landsmeer, The Netherlands) as a secondary antibody. Protein expression was detected with the ChemiDoc™ XRS, analyzed by Quantity One (Biorad, Veenendaal, The Netherlands) and adjusted for total protein levels per lane, using coomassie brilliant blue staining.

### Mitochondrial DNA content

Nuclear and mitochondrial DNA (mtDNA) was isolated from RP WAT with the QIAamp DNA micro kit (Qiagen Benelux b.v., Zwijndrecht, The Netherlands), according to manufacturers protocol. DNA quantity was determined with a Nanodrop 2000 (Thermo Scienctific, Breda, The Netherlands). Relative mitochondrial DNA expression was determined as previously described [33]. Briefly, 135 ng input DNA was used for each qPCR reaction, with NADH dehydrogenase 1 (*Nd1*) as a marker for mitochondrial DNA and *Lpl* to normalize for nuclear DNA. Primers sequences are shown in Table 2. Data were analyzed using qbase^+^ (Biogazelle, Genth, Belgium).

### Statistical analysis

Statistical analyses were performed using SPSS® 19.0 (SPSS Benelux, Gorinchem, The Netherlands). The primary focus for the study was to assess the difference between the Concept and the CTRL group, which was tested using a TTest. The latter will give insight into the window of opportunity to be influenced by our nutritional intervention as well as to understand the direction of change. The effect of the WSD challenge was tested separately using a TTest. Concept and REF group were not directly compared based on the differences in dietary exposure both in early postnatal life (Concept vs. Control) and also later life (WSD vs. AIN). Gaussian distribution was tested with a Kolmogorov-Smirnov test. Differences in adipocyte size distribution were analyzed with a Mann-Whitney. Correlations between parameters were analyzed using a Pearson test. Data are presented as mean + SD, except for the qPCR data which are presented as mean relative expression (scaled to the average expression) with 95% confidence intervals. Differences were considered significant at *p* < 0.05 and tendencies reported when 0.05 < *p* < 0.1.

## Results

### Effect of the western-style diet challenge during adolescence and adulthood

#### Body composition

As a consequence of the WSD challenge, body weight, fat mass, EPI and RP WAT weight were increased in the CTRL compared to the non-challenged REF group. Lean body mass was slightly higher although no significant effect on *M. tibialis* weight was found (Table 3). These data are discussed elsewhere [20].

**Table 3:** Body composition, organ weight and EPI WAT adipocyte characteristics (mean adipocyte size and number)

Diet group	REF (*n* = 12)	CTRL (*n* = 12)	Concept (*n* = 12)
Body weight (g)	28.5 (SD 1.8)	31.7 (SD 2.5) ^##^	29.2 (SD 2.4) *
Lean body mass (g)	23.5 (SD 1.4)	24.4 (SD 1.3) †	23.5 (SD 1.8)
Fat mass (g)	4.9 (SD 1.1)	7.5 (SD 1.8) ^###^	5.8 (SD 1.6) *
RP WAT (g)	0.14 (SD 0.06)	0.31 (SD 0.10) ^###^	0.20 (SD 0.10) *
EPI WAT (g)	0.48 (SD 0.15)	1.00 (SD 0.29) ^###^	0.63 (SD 0.27) **
*M. tibialis* (g)	0.05 (SD 0.00)	0.05 (SD 0.01)	0.05 (SD 0.01)
Adipocyte characteristics:	REF (*n* = 11)	CTRL (*n* = 10)	Concept (*n* = 9)
Mean adipocyte size (μm)	74.4 (SD 10.3)	107.1 (SD 15.7) ^###^	81.3 (SD 14.0) **
Adipocyte number (× 10^6^)	7.44 (SD 2.44)	5.79 (SD 1.07) †	7.55 (SD 3.67)

Data expressed as mean plus SD. Early diet and WSD challenge effects were analyzed separately. Difference between Concept and REF groups were not tested, as groups were fed different postnatal and adult diets. ##: *p* < 0.01; ###: *p* < 0.001; †: *p* = 0.05–0.10, CTRL different from REF group; *: *p* < 0.05; **: *p* < 0.01, Concept different from CTRL group

#### Adipocyte size and number in EPI WAT

**Fig. 2:**
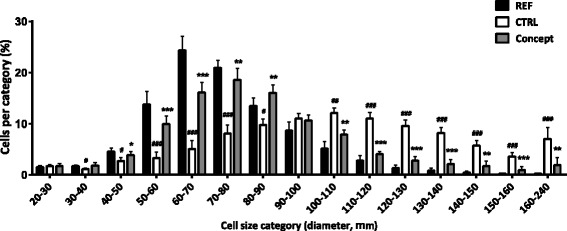
Effect of postnatal Concept diet on adult adipocyte size distribution of epididymal white adipose tissue (*n* = 10, *n* = 9 and *n* = 11 for CTRL, Concept and REF group respectively). Early diet and WSD challenge effects were analyzed separately. Difference between Concept and REF group not tested, as groups were fed different postnatal and adult diets. #: *p* < 0.05; ##: *p* < 0.01; ###: *p* < 0.001, CTRL different from REF group; *: *p* < 0.05; **: *p* < 0.01; ***: *p* < 0.001 Concept different from CTRL group

The WSD challenge resulted in a 44% increase in average EPI adipocyte size in the CTRL group compared to the non-challenged REF group (*p* < 0.001; Table 3). Furthermore, a shift was seen in adipocyte size distribution towards larger cells in the CTRL group (Fig. 2). Percentages of cells in the size categories between 30 and 90 μm were lower for the CTRL group while higher in the size categories between 100 and 240 (*p* < 0.05). EPI adipocyte numbers tended to be lower in the CTRL group (*p* = 0.07; Table 3).

#### Markers for mitochondrial content and β-oxidation in the WAT

**Fig. 3:**
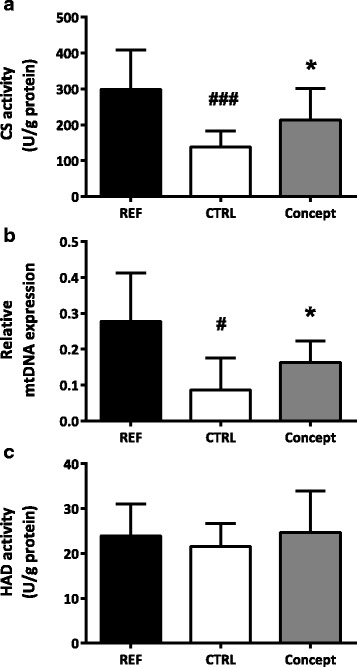
Effect of postnatal Concept diet on markers for mitochondrial content and β-oxidation in the retroperitoneal (RP) white adipose tissue; (**a**) citrate synthase (CS) activity (*n* = 10 per group), (**b**) mtDNA/nDNA ratio (*n* = 8 per group) and (**c**) hydroxyacyl-CoA dehydrogenase (HAD; *n* = 10 per group). DNA ratio displayed as mean plus 95% confidence interval. Early diet and WSD challenge effects were analyzed separately. Difference between Concept and REF group not tested, as groups were fed different postnatal and adult diets. #: *p* < 0.05; ###: *p* < 0.001, CTRL different from REF group; *: *p* < 0.05, Concept different from CTRL group

Mitochondrial content of the RP depot, as measured by CS activity and relative mtDNA content, was significantly lower in the CTRL group compared to the REF group (*p* < 0.05; Fig. 3a and b). FA oxidation of the RP WAT, reflected by HAD activity, was similar in both groups (Fig. 3c).

#### OXPHOS protein expression in the WAT

**Fig. 4:**
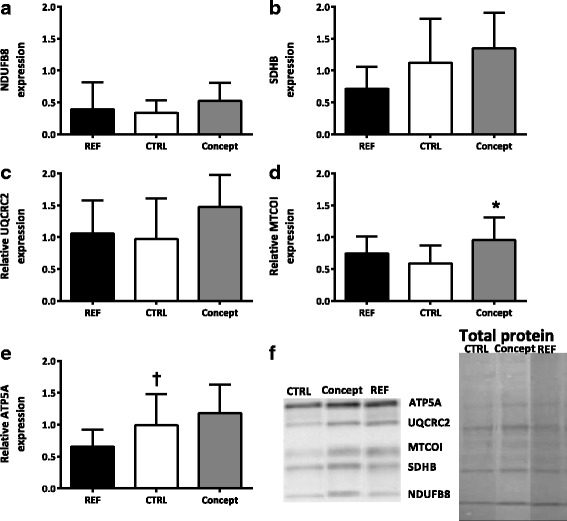
Effect of postnatal Concept diet on relative protein expression of 5 oxidative phosphorylation complex (OXPHOS) subunits (**a** – **e**) in the retroperitoneal (RP) white adipose tissue (*n* = 8 for CTRL, *n* = 7 for Concept and *n* = 10 for REF group) and (**f**) total protein and western blot bands of one sample per group, samples came from one blot, but not from adjacent lanes, a picture of the whole blot is added as Additional file 1. Early diet and WSD challenge effects were analyzed separately. Difference between Concept and REF group not tested, as groups were fed different postnatal and adult diets. *: *p* < 0.05, Concept different from CTRL group; †: *p* 0.05–0.1 REF different from CTRL group

**Fig. 5:**
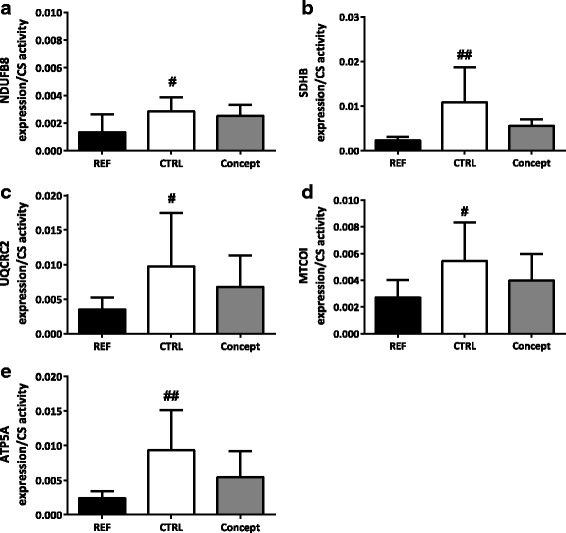
Effect of postnatal Concept diet on relative protein expression of 5 oxidative phosphorylation complex (OXPHOS) subunits (**a** - **e**) in the retroperitoneal (RP) white adipose tissue when corrected for citrate synthase (CS) activity (*n* = 7 for CTRL, *n* = 6 for Concept and *n* = 9 for REF group). Early diet and WSD challenge effects were analyzed separately. Difference between Concept and REF group not tested, as groups were fed different postnatal and adult diets. #: *p* < 0.05; ##: *p* < 0.01 different from CTRL group

No effect of the WSD challenge was shown on the protein expression of OXPHOS complexes I (NDUFB8; Fig. 4a), II (SDHB; Fig. 4b), III (UQCRC2; Fig. 4c) and IV (MTCOI; Fig. 4d) in the RP depot. Expression levels of the ATP synthase subunit of complex V (ATP5A) tended to be increased in the CTRL group as a consequence of the WSD challenge (*p* = 0.08; Fig. 4e). OXPHOS protein expression was lower for all complexes in the CTRL compared to the REF group when corrected for CS activity (Fig. 5a-e).

#### Gene expression profiles RP WAT

Expression levels of the lipogenic genes *Gpat* and *Srebp1c* in RP WAT were lower in the CTRL compared to the REF group as were the expression levels of the lipogenic enzymes *Acc1*, *Fas* and *Scd1* (*p* < 0.05 for all parameters; Table 4). Gene expression of the lipolytic enzyme *Atgl* tended to be lower in the CTRL group (*p* = 0.09). Expression of *Hsl* was similar in both groups. Expression levels of *Pdk4* and *HkII*, both part of the glucose oxidative pathway, were significantly lower in the CTRL compared to the REF group as were the levels of the glucose transporter *Glut4* (*p* < 0.05 for all parameters; Table 4). The CTRL group showed elevated expression levels of the FA transporters *Cpt1α* and *Cd36* and lower *Fatp1* and *Fabp4* levels compared to the non-challenged REF group (*p* < 0.05 for all parameters). No difference was observed in expression levels of LPL (Table 4). *Pparα* expression levels were lower in the CTRL group compared to the REF group (*p* < 0.001), as was the UCP3 gene expression (*p* < 0.01; Table 4). Expression of WAT browning markers Ucp1 and Cidea were decreased in the CTRL compared to the Ref group (*p* < 0.001; Table 4).

**Table 4:** Relative mRNA expression in arbitrary units (AU)

Gene name	REF (*n* = 10)	CTRL (*n* = 12)	Concept (*n* = 12)
RP WAT:			
*Acc1*	2.953 (1.912–4.562)	1.000 (0.747–1.338) ^###^	1.212 (0.815–1.803)
*Atgl*	1.264 (1.118–1.429)	1.000 (0.786–1.273) †	1.058 (0.831–1.347)
*Cd36*	0.863 (0.741–1.006)	1.000 (0.939–1.064) ^#^	0.882 (0.764–1.018) ‡
*Cidea*	10.066 (6.317–16.038)	1.000 (0.603–1.659) ^###^	1.962 (1.035–3.717) ‡
*Cpt1α*	0.711 (0.595–0.849)	1.000 (0.816–1.225) ^#^	0.978 (0.764–1.251)
*Fabp4*	1.199 (1.020–1.408)	1.000 (0.892–1.121) ^#^	1.097 (1.002–1.200)
*Fas*	3.012 (1.816–4.995)	1.000 (0.730–1.371) ^#^	1.080 (0.715–1.633)
*Fatp1*	1.352 (1.097–1.665)	1.000 (0.821–1.218) ^#^	0.995 (0.804–1.230)
*Glut4*	1.939 (1.315–2.858)	1.000 (0.863–1.159) ^###^	1.221 (0.935–1.594)
*Gpat*	1.514 (1.298–1.766)	1.000 (0.846–1.181) ^###^	1.167 (0.925–1.472)
*HkII*	1.390 (1.093–1.767)	1.000 (0.786–1.272) ^#^	1.068 (0.795–1.434)
*Hsl*	1.146 (1.048–1.252)	1.000 (0.837–1.194)	0.931 (0.809–1.071)
*Lpl*	0.902 (0.784–1.038)	1.000 (0.874–1.144)	0.866 (0.706–1.061)
*Pdk4*	1.878 (1.215–2.904)	1.000 (0.780–1.283) ^##^	1.377 (0.887–2.140)
*Pparα*	1.911 (1.648–2.216)	1.000 (0.785–1.274) ^###^	1.124 (0.944–1.338)
*Scd1*	1.891 (1.544–2.317)	1.000 (0.812–1.232) ^###^	1.079 (0.854–1.363)
*Srebp1c*	1.426 (1.146–1.775)	1.000 (0.810–1.235) ^##^	1.087 (0.910–1.300)
*Ucp1*	27.480 (15.828–47.709)	1.000 (0.337–2.968) ^###^	2.621 (0.860–7.993)
*Ucp3*	1.432 (1.248–1.644)	1.000 (0.833–1.200) ^##^	1.168 (0.883–1.544)
Skeletal muscle:	REF (*n* = 10)	CTRL (*n* = 12)	Concept (*n* = 11)
*Cpt1a*	1.029 (0.881–1.202)	1.000 (0.752–1.331)	1.074 (0.906–1.273)
*Glut4*	1.009 (0.811–1.256)	1.000 (0.811–1.233)	1.089 (0.939–1.262)
*Pdk4*	1.600 (1.124–2.277)	1.000 (0.684–1.461)	1.296 (0.929–1.808)
*Ucp3*	1.577 (1.286–1.933)	1.000 (0.830–1.204) ^##^	1.299 (1.105–1.527) *

mRNA expression listed as the mean expression level, scaled to expression of the CTRL group, plus 95% confidence intervals, *n* = 10–12 per group. Early diet and WSD challenge effects were analyzed separately. Difference between the Concept and REF group were not tested, as groups were fed different postnatal and adult diets. #: *p* < 0.05; ##: *p* < 0.01; ###: *p* < 0.001; †: *p* = 0.05–0.10, CTRL different from REF group; *: *p* < 0.05; ‡: *p* = 0.05–0.10, Concept different from CTRL group

#### Gene expression profiles in skeletal muscle

The CTRL group showed lower *Ucp3* expression levels in the *m. tibialis* compared to the REF group (*p* < 0.01). Expression profiles of *Pdk4*, *Cpt1α* and *Glut4* were not affected by the adult WSD challenge (Table 4)*.*

### Programming effect of concept IMF diet

#### Body composition

In accordance to previous findings [18, 19], body weight, fat mass, EPI and RP WAT weight were reduced in the Concept compared to the CTRL group. Lean body mass and *m. tibialis* weight were similar in both groups (Table 3). These data are part of a more extensive study concerning the potential protective effects of structural aspect of lipids in human milk against later life obesity and therefore reported in a separate paper [20].

#### Adipocyte size and number in EPI WAT

Average EPI adipocyte size (Table 3) was 24% lower in the Concept group compared to the CTRL group (*p* < 0.01). This was confirmed by a shift in adipocyte size distribution towards smaller cells in the Concept compared to the CTRL group (Fig. 2). Most striking differences were found in size categories between 50 and 90 μm with more cells for the Concept compared to the CTRL group (*p* < 0.05). In addition, the Concept group showed fewer cells in the size categories between 100 and 240 μm (*p* < 0.01) compared to the CTRL group. EPI adipocyte numbers were similar in both groups (Table 3).

#### Markers for mitochondrial content and β-oxidation in WAT

In the RP depot mitochondrial content, as measured by CS activity and relative mtDNA expression, was significant higher in the Concept compared to the CTRL group (*p* < 0.05; Fig. 3a and b respectively). Groups had similar HAD activity, which reflects FA oxidation (Fig. 3c).

#### OXPHOS protein expression in WAT

Mitochondrial cytochrome c oxidase I subunit (subunit of complex IV, MTCOI) expression levels were significantly higher in the Concept group compared to the CTRL group (*p* < 0.05; Fig. 4d). Protein expression of complex I (NDUFB8; Fig. 4a) II (SDHB; Fig. 4b), III (UQCRC2, Fig. 4c) and V (ATP5A; Fig. 4e) did not significantly differ between the two groups. OXPHOS protein expression was not significantly lower in the Concept compared to the CTRL group when corrected for CS activity (Fig. 5a-e).

#### Gene expression profiles in RP WAT

RP WAT expression levels of lipogenic genes *Gpat*, *Srebp1c, Acc1*, *Fas* and *Scd1* were similar in the Concept and the CTRL group as were the expression levels of lipolytic enzymes *Atgl* and *Hsl* (Table 4).

Glucose oxidative pathways (PDK4 and HKII) were unaffected by the early diet (Table 4). The *Cd36* expression levels of the Concept group tended to be lower compared to the CTRL group (*p* = 0.09), but expression levels of other FA transporters (*Cpt1α, Fatp1, Fabp4* and *Lpl*) and glucose transporter *Glut4* were comparable between the Concept and the CTRL group (Table 4). *Pparα* (fat oxidative pathway) expression levels were also similar in the Concept and CTRL group as was the RP WAT expression of the uncoupling protein *Ucp3* (Table 4). Expression of *Ucp1*, as marker for WAT browning, was similar between the Concept and CTRL group, but expression of another marker for WAT browning, *Cidea* tended to be higher in Concept compared to CTRL group (*p* = 0.08; Table 4).

#### Gene expression profiles in skeletal muscle

The Concept group showed higher *Ucp3* expression levels in the *m. tibialis* compared to the CTRL group (*p* < 0.05; Table 4). Expression profiles of *Pdk4* (involved in the transport of pyruvate –the glycolytic end product- into the mitochondria), *Cpt1α* (involved in the transport of long-chain fatty acids over the mitochondrial membrane) and *Glut4* (glucose transporter) in the *m. tibialis* were not affected by the early postnatal diet (Table 4).

#### Correlations

**Fig. 6:**
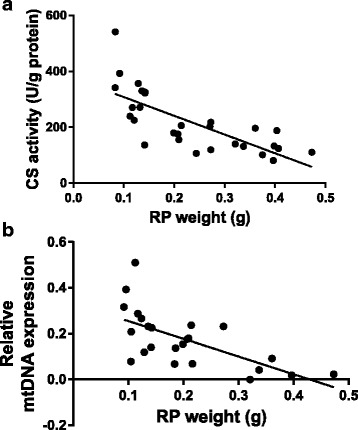
Correlation between retroperitoneal (RP) white adipose tissue weight and (**a**) citrate synthase (CS) activity (*r* = −0.741; *p* < 0.001) and (**b**) mtDNA/nDNA ratio(*r* = −0.655; *p* < 0.001)

Markers for mitochondrial content, CS activity and mtDNA, were inversely correlated to RP WAT weight (*r* = −0.741; *p* < 0.001 and *r* = −0.655; *p* < 0.001, respectively; Fig. 6). No correlations between protein expression of the OXPHOS complexes and RP WAT weight were found.

## Discussion

The present study showed that early life exposure to large, phospholipid-coated lipid droplets leads to altered expression of markers for mitochondrial oxidative capacity in RP WAT and *m. tibialis*. Although functional mitochondrial capacity was not measured, these markers suggest that adapted mitochondrial oxidative capacity may underlie the previously reported reduced body fat accumulation in adolescence and adulthood [18–20].

Programming of metabolic health is well established, but possible underlying mechanisms are still largely unknown although many have been suggested. Aberrant mitochondrial function has been suggested in a limited amount of studies as possible link between adverse fetal environment and the development of T2D [22, 34]. Yet, little is known on how nutritional programming may improve lifelong metabolic health and protect against obesity.

In the present study, markers for mitochondrial oxidative capacity appeared to be increased due to the supramolecular structure of milk lipids in the early postnatal diet. The elevation in CS activity and relative mtDNA expression in the WAT imply higher mitochondrial content, suggesting a higher oxidative capacity. In addition, the elevated protein expression levels for the mitochondrial cytochrome c oxidase subunit I indicate that possibly more substrate is oxidized in RP WAT of Concept mice as well. As OXPHOS protein levels corrected for CS activity were similar between CTRL and Concept groups, total oxidative capacity per cell may be increased by the Concept diet rather than the intrinsic activity per mitochondrion. The expression levels of *Ucp3* in the Concept group were slightly increased in skeletal muscle. A higher *Ucp3* expression could elevate substrate oxidation via its uncoupling activity or via export of FA anions out of the mitochondria [35, 36]. Taken together, these data suggest that mitochondrial oxidative capacity may be elevated in mice fed a Concept diet in early postnatal life, resulting in a better ability to cope with an increased FA load in adulthood, which could explain the lower adiposity seen upon the WSD challenge.

The WSD challenge had, apart from the early diet, a clear effect on markers for oxidative capacity. Expression of markers for mitochondrial content (CS activity and relative mtDNA expression) in the WAT and mitochondrial uncoupling (*Ucp3*) in the skeletal muscle were decreased in the CTRL compared to the non-challenge REF group. No effect of the WSD diet on OXPHOS protein levels was found. OXPHOS protein levels corrected for CS activity were however elevated in the CTRL compared to the REF group, which points towards increased intrinsic activity per mitochondrion as compensatory mechanism. These results are in line with the literature showing decreased expression of oxidative capacity markers in the WAT upon a high fat diet [37] and are opposite from the early diet effect. This may indicate that mice fed the Concept diet in early life were protected against the adverse effects of the WSD challenge by preserving WAT health and were different from the CTRL mice, able to increase mitochondrial oxidative capacity in response to the WSD challenge.

Gene expression of markers for lipogenesis, lipolysis or lipid uptake were unaffected by the supramolecular lipid structure of the early postnatal diet, suggesting that these metabolic processes may not contribute the observed effects on adult adiposity. In addition, gene expression of markers for β-oxidation, transfer of long-chain acyl-CoA into the mitochondria, other substrate transporters, the glucose oxidative pathway and browning of the WAT depot were not affected by the postnatal diet intervention.

It should be noted that functional measurements are pivotal to confirm whether the higher levels of mitochondrial content and capacity markers truly result in increased substrate oxidation upon a change in nutrient status. There is some evidence showing that mitochondrial function can be reduced or elevated upon caloric restriction and/or physical activity with similar numbers of mitochondria [38, 39], indicating that similar numbers of mitochondria still may result in differences in mitochondrial function. In contrast, other studies have shown that mitochondrial content and complex IV protein content and activity were correlated to ADP-stimulated respiration [40–42], suggesting that differences in levels of these markers may reflect an actual difference in mitochondrial function. Thus, although functional measurement were not performed, the lower adiposity and adipocyte size of the Concept group found, does suggest that the Concept mice showed a less exaggerated respond to the WSD challenge. This supports oxidation of the surplus of energy instead of storage into WAT.

Altered mitochondrial oxidative capacity in WAT could affect substrate selection and competition in other oxidative organs like skeletal muscle and liver, thereby affecting whole body insulin sensitivity [43, 44]. Further studies focusing on functional measurements of mitochondrial function in liver and skeletal muscle are required to confirm that postnatal dietary lipid structure indeed programs adult mitochondrial function.

CS activity and mtDNA content, representative for mitochondrial content, were inversely correlated with RP WAT weight. Whether or not the higher mitochondrial content in the Concept group caused the reduced RP WAT weight or the other way around remains to be determined. Interestingly cytochrome c oxidase subunit I expression was not related to RP WAT weight, suggesting that OXPHOS protein expression may be driven by the interaction between the early diet and the WSD challenge although further investigation is needed to establish this.

Previous studies showed that mitochondrial oxidative capacity was decreased in rodents and sheep by exposure to HFD and/or malnutrition during fetal life [45–47]. The present results show a beneficial programming of mitochondrial oxidative capacity markers by a relatively mild diet intervention during the postnatal period. Based on these observations we could speculate that decreased oxidative capacity following an adverse fetal environment could potentially be reprogrammed by an adjusted diet in the postnatal period.

## Conclusion

We showed that exposure to adapted supramolecular structure of dietary lipids (Nuturis®) in early postnatal life results in increased expression of markers for mitochondrial content and capacity which may enable adult mice to cope better with the surplus of fat provided by the WSD challenge diet. Although functional measurements are not performed, these adaptations in mitochondrial function markers could underlie the observed reduced body fat accumulation in an obesogenic adult environment in these animals.

## Additional file

Additional file 1: Figure S1. OXPHOS western blot. CTRL: sample of CTRL group; Conc: sample of Concept group; REF: sample of REF group; p.c.: positive control sample. (PDF 85 kb)

## Abbreviations

*18SrRNA*: 18S ribosomal RNA
*Acc1*: Acetyl-CoA carboxylase 1
AIN93G: American Institute of Nutrition-93G synthetic diet
*Atgl*: Adipose triglyceride lipase
ATP5A: ATP synthase subunit of complex V of oxidative phosphorylation
*Canx*: Calnexin
*Cd36*: Fatty acid transfer protein/cluster of differentiation 36
*Cpt1a*: Carnitinepalmitoyl-transferase 1a
CS: Citrate synthase
EPI: Epididymal fat depot
FA: Fatty acids
*Fabp4*: Fatty acid binding protein 4
*Fasn*: Fatty acid synthase
*Fatp1*: Fatty acid transfer protein 1
*Glut4*: Glucose transporter 4
*Gpat*: Glycerol-3-phosphate acyltransferase
HAD: Hydroxyacyl-CoA dehydrogenase
HFD: High fat diet
*HkII*: Hexokinase II
*Hsl*: Hormone sensitive lipase
IMF: Infant milk formula
*Lpl*: Lipoprotein lipase
MTCOI: Mitochondrial encoded cytochrome C oxidase I subunit of complex IV of oxidative phosphorylation
MtDNA: Mitochondrial DNA
*Nd1*: NADH dehydrogenase 1
NDNA: Nuclear DNA
NDUFB8: NADH dehydrogenase 1 beta subunit of complex I of oxidative phosphorylation
OXPHOS: Oxidative phosphorylation complex
*Pdk4*: Pyruvate dehydrogenase kinase 4
PN: Postnatal day
*Pparα*: Peroxisome proliferator activated receptor α
RP: Retroperitoneal fat depot
*Rpl19*: Ribosomal protein L19
*Rps29*: Ribosomal protein S29
*Scd1*: Stearoyl-Coenzyme A desaturase 1
SDHB: Succinate dehydrogenase subunit of complex II of oxidative phosphorylation
*Srebp1c*: Sterol regulatory element binding protein 1c
T2D: Type 2 diabetes
*Ucp*: Uncoupling protein
UQCRC2: Ubiquinol cytochrome C reductase core protein subunit III of oxidative phosphorylation
WAT: White adipose tissue
WSD: Western style diet
