# Supplementary material for: Supramolecular structure of dietary fat in early life modulates expression of markers for mitochondrial content and capacity in adipose tissue of adult mice
Source: Nutr Metab (Lond). 2017 Jun 12;14:37. doi: 10.1186/s12986-017-0191-5 (PMC5469001; doi:10.1186/s12986-017-0191-5)
Supplement: Additional file 1: Figure S1. — OXPHOS western blot. CTRL: sample of CTRL group; Conc: sample of Concept group; REF: sample of REF group; p.c.: positive control sample. (PDF 85 kb) [file 12986_2017_191_MOESM1_ESM.pdf]

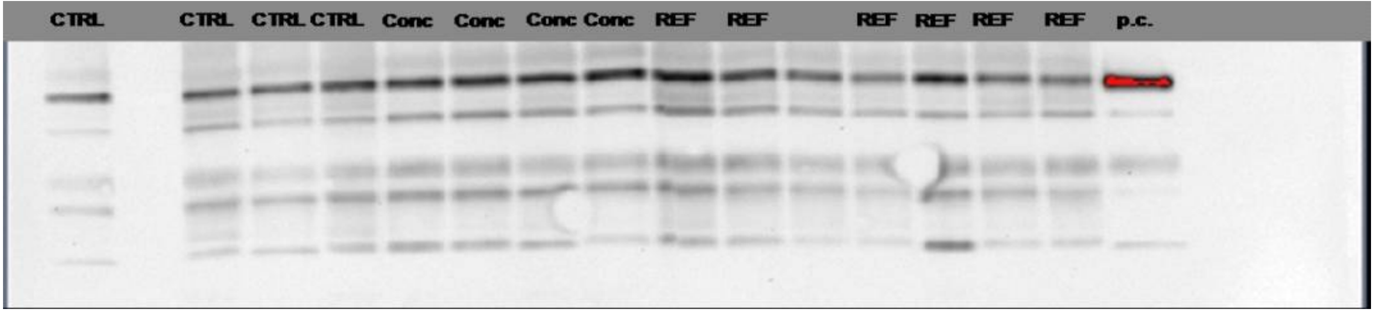

Figure S1: OXPHOS western blot. CTRL: sample of CTRL group; Conc: sample of Concept group; REF: sample of REF group; p.c.: positive control sample.
